# Experiment Study on Rock Mass Classification Based on RCM-Equipped Sensors and Diversified Ensemble-Learning Model

**DOI:** 10.3390/s24196320

**Published:** 2024-09-29

**Authors:** Feng Li, Huike Zeng, Hongbin Xu, Haokai Sun

**Affiliations:** 1 College of Civil and Transportation Engineering, Shenzhen University, Shenzhen 518060, China; lf260986@semi.ac.cn (F.L.); xuhongbin@semi.ac.cn (H.X.); 2 National Key Laboratory of Green and Long-Life Road Engineering in Extreme Environment (Shenzhen), Shenzhen University, Shenzhen 518060, China; 3 School of Qilu Transportation, Shandong University, Jinan 250061, China; zeng_huike@163.com; 4 Geotechnical and Structural Engineering Research Center, Shandong University, Jinan 250061, China

**Keywords:** TBM-equipped sensor, rock mass classification, stacking ensemble-learning model, multi-source data

## Abstract

The geological condition monitoring and identification based on TBM-equipped sensors is of great significance for efficient and safe tunnel construction. Full-scale rotary cutting experiments are carried out using tunnel-boring machine disc cutters. Thrust, torque and vibration sensors are equipped on the rotary cutting machine (RCM). A stacking ensemble-learning model for real-time prediction of rock mass classification using features of mathematical statistics is proposed. Three signals, thrust, torque and a novel vibration spectrogram-based local amplification feature, are fed into the model and trained separately. The results show that the stacked ensemble-learning model has better accuracy and stability than any single model, showing a good application prospect in the rock mass classification.

## 1. Introduction

According to the requirements of safe and rapid excavation and the improvement of underground engineering technology, the tunnel-boring machine (TBM) has been widely used and rapidly developed [1]. The drilling and blasting methods still rely on manual, mechanical, or blasting, followed by the construction of a support structure based on the surrounding rock conditions. Many construction procedures, such as excavation, ballast removal, and support, result in a slow excavation speed, high labor intensity, and low safety, especially in urban underground space projects [2,3]. According to the requirements of safe and rapid excavation and the improvement of underground engineering technology, the tunnel-boring machine (TBM) has been widely used and rapidly developed. TBM can simultaneously perform multiple steps and has many advantages, such as fast excavation speed, precise deformation control, safety, and environmental protection [4]. It has been widely applied in constructing railways, highways, subways, water conservancy, coal, and other fields [5].

Tunnel engineering is gradually developing in the direction of large sections and long distances. When using TBM for tunnel excavation, the geological conditions are complex, and there are many risk sources [6]. There are complex landscapes with severe fluctuations and unfavorable geological conditions [7], such as a large burial depth, extremely hard rock, a strong rock burst [8], a soft and large deformation, a mud and water inrush, and a high geothermal energy. However, TBM is sensitive to changes in complex geological conditions [9]. If the operating parameters are not adjusted in real time, abnormal wear of the cutter and tool can easily occur. These issues can result in inefficient excavation and higher construction costs. Therefore, accurate measurement of the geological and main rock mass parameters before TBM tunneling is particularly important for efficiency and safety [10]. The full-scale rotary cutting machine (RCM) is the most commonly used test rig. Shin [11] simulated the excavation process of a hard rock tunnel-boring machine (TBM) and conducted tests on granite using different disc-cutter sizes. Zhang [12] carried out rock fragmentation tests under different rolling radii. Geng [13] conducted cutting experiments on rocks with different inclination angles and thicknesses. However, the rock classification method based on different tool installation positions and the dynamic response of components such as tools, cutterheads, and rock masses requires further experimental research.

Before construction, the geological conditions along the tunnel can be roughly obtained through geological exploration [14]. Many approaches have been applied to study the geological prediction in front of TBM tunnel excavation, including core testing, seismic waves [15], microseismic [16], and resistivity. However, these methods roughly predict the strength and integrity of the rock mass in a certain section or the larger-scale undesirable geology. In addition, they can only be performed during the excavation stoppage, as they are unable to provide the real-time and accurate perception of rock mass parameters during excavation. Therefore, it is very important to propose a method that can accurately predict the classification of the rock mass in real time.

Many researchers have introduced machine-learning methods to evaluate rock mass quality through these monitoring data [17]. With these methods, the relationship between operational data and rock mass classification can be characterized [18,19], minimizing the subjectivity and inaccuracy of artificial evaluation [20]. Currently, the most widely used algorithms are the long short-term memory network (LSTM) [21] and ensemble-learning algorithm [22]. Ayawah [23] evaluated the possibility of a single machine-learning model to predict ground conditions or rock mass in front of TBMs. Liu [24] proposed a hybrid algorithm that integrates the backpropagation neural network with simulated annealing to predict the rock mass parameters based on TBM drive parameters. Santos [25] used multivariate statistics and artificial intelligence to predict the Rock Mass Rating (RMR) classification index. A novel method for rock mass classifications was proposed, reducing subjectivity in the parameters and classification methods. Zhang [26] proposed a generative adversarial network for geological prediction, which accurately estimated the thickness of each rock–soil type in a TBM construction tunnel. In order to establish prediction models, Xu [27] evaluated the application of five different statistical and ensemble machine-learning methods and two different deep neural networks. It was proved that the accurate prediction of the advance rate, rotation speed, thrust, and torque indicators based on the operating parameters could guide the control and application of a TBM. Hou [28] selected ten key operation parameters for prediction. The results indicated that the stacking ensemble classifier performs better than individual classifiers, exhibiting more powerful learning and a generalization ability for small and imbalanced samples. Most previous studies are based on a single machine-learning model [29] and less meaningful features for rock-mass parameter sensing, resulting in a single algorithm function with high limitations and low accuracy.

This paper aims to build a rock mass classification model with high accuracy and stability. The remainder of this paper is organized as follows: Section 2 presents the framework and methodology. The experimental procedure and data analysis are described in Section 3. Section 4 introduces the construction of the stacking technique for ensemble learning. Section 5 gives the results and discussion. The conclusions are in Section 6.

## 2. Materials and Methods

**Figure 1:**
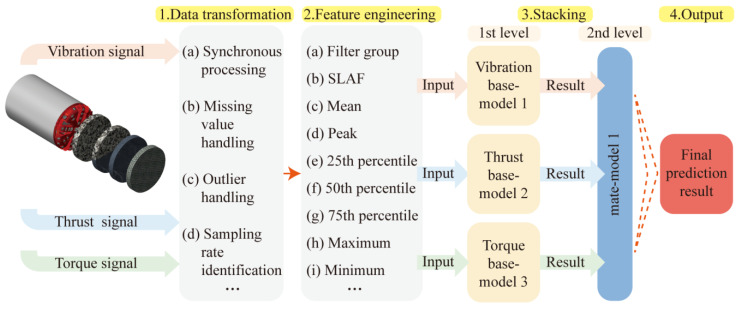
Structure of a rock mass strength-forecasting model.

This section presents the methodology of the modeling process of the rock mass classification forecasting employed in this paper. It primarily includes five parts: data transformation, feature engineering, stacking ensemble model construction, and model evaluation. The frame diagram in Figure 1 illustrates the implementation process of the proposed model.

First, through a large number of literature surveys, it is determined that the TBM signal types used this time are highly correlated with rock mass types, namely thrust [25], torque [30], and vibration. However, the direct use of time-domain signals will result in a tedious and time-intensive computational process. Therefore, feature engineering is performed on the three signals separately. The time-domain signal is refined into feature vectors as input to the algorithm. Then, the modeling process of the stacking technique of ensemble learning is introduced. Finally, metrics such as accuracy, confusion matrix, and stability are introduced.

### 2.1. Data Transformation

In the signal acquisition, the magnitude of vibration, thrust, and torque are collected and transmitted by wireless acceleration sensors, thrust sensors, and torque sensors, respectively. In this process, outliers and missing values inevitably occur. Therefore, the three time-domain signals are synchronously processed. Missing values are filled by interpolation, and fragments containing outliers are removed. The timestamp node is defined by the time for each revolution of the cutterhead, and the three signals are truncated and saved synchronously. Data from the initial rising stage and the unloading section were excluded. Only the table segment is retained as the analysis object for rock mass-classification prediction.

### 2.2. Feature Engineering Based on TBM Parameters

This section introduces the basics of feature engineering for text. The input length is closely related to the computation time of the machine-learning model, and a redundant input of signals can also lead to a decrease in accuracy. Therefore, it is crucial to design features to describe the relationship between rock mass and algorithms, making it possible to evaluate and optimize the stacking technique for ensemble learning.

#### 2.2.1. Torque and Thrust

With reference to signal analysis methods and mathematical and statistical fundamentals, 9 characteristic of torque and thrust signals were calculated, respectively: maximum (Max), minimum (Min), peak, mean, variance (Var), root mean square (RMS), 25th percentile (Q1, the position is *P_Q_*_1_), 50th percentile (Q2, the positions is *P_Q_*_2_), and 75th percentile (Q3, the positions is *P_Q_*_3_). Its calculation formula is shown in Table 1.

#### 2.2.2. Vibration

**Figure 2:**
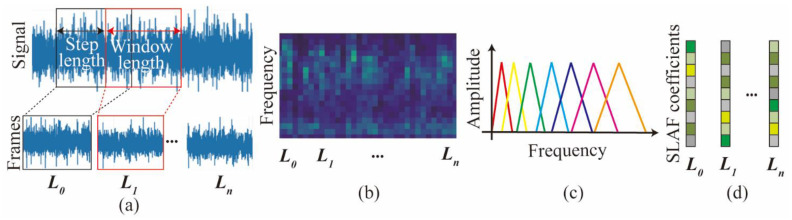
Schematic flow for SLAF. (**a**) windowing and framing; (**b**) Time spectra; (**c**) Filter banks; (**d**) Cepstral coefficients.

In this section, a feature engineering method is proposed to convert the vibration time-domain signal into a novel spectrogram-based local amplification feature (SLAF). The processing flow is shown in Figure 2.

In this study, to prevent spectral leakage, the Hanning window is used for windowing. The fundamental idea of the Hanning window is to gradually taper the data at the end of the record, and therefore to avoid the abrupt truncation by a rectangular window [30]. The Hanning window can be expressed as Equation (1). Windowing and time segmentation are used for transforming signals to frames. In this paper, a 50% window-width step length and a window length of 1 s are used.

(1) $$w_{\left(t\right)}=0.5-0.5\cos\left[\frac{2\pi \left(t+1\right)}{T+1}\right],\;0\leq t\leq T-1$$

where *t* is the time, and *T* is the window width.

Further, Fast Fourier Transform (FFT) is performed on each frame after division and windowing. This converts the vibration signal from the time domain to the frequency domain signal (*X_a_*(*k*)). And the frequency of each frame in the time dimension is superimposed to obtain a spectrogram. It represents the frequency characteristics of the vibration of the cutterhead over a period of time. The *X_a_*(*k*) is as follows:

(2) $$X_{a}\left(k\right)=\sum_{n=0}^{N-1}x(n)e^{-j2\pi \mathrm{nk}/N}\;0\leq k\leq N$$

where *a* is *a*th frame, *k* represents the *k*th spectral line in the frequency domain, and N is the number of sampling points.

The main frequency of the cutterhead vibration is concentrated in the local range. It represents that the perception of frequency is non-linear. Therefore, a triangular filter bank is used, and the layout is sparse and adjustable. Only filter banks with the same bandwidth are shown in Figure 2c. Through the operation of spectrum and filter bank, the low-dimensional feature in the range of each filter bank can be obtained, as shown in Figure 2d. This preserves the frequency integrity, while also simplifying the input feature dimension of the original features. The expression function of the triangular filter is as follows:

(3) $$H_{m}\left(k\right)=\left\{\begin{array}{cc} 0 & k<f(m-1)\\ \frac{k-f(m-1)}{f\left(m\right)-f\left(m-1\right)} & f(m-1)\leq k\leq f(m)\\ \frac{f\left(m+1\right)-k}{f\left(m+1\right)-f(m)} & f(m)\leq k\leq f(m+1)\\ 0 & k>f(m+1) \end{array}\right.$$

where *H*_m_(*k*) is the *k*th value of the *m*th filter in the filter bank, and *f*(*m*) is the corresponding frequency.

The energy (*E*(m)) in the range of a single filter can be obtained by calculating the spectrum and filter bank. After dividing by the frequency length of this filter bank, it is expressed as the mean vibrational spectral energy. The *E*(m) can be expressed as follows:

(4) $$E\left(m\right)=\frac{\sum_{k=0}^{N-1}{\left|X_{a}\left(k\right)\right|}^{2}H_{m}(k)}{f\left(m+1\right)-f(m-1)}$$

#### 2.2.3. TBM Performance

The advance rate (AR, mm/min), rotating speed (RS, rev/min), field penetration index (*FPI*, kN/cutter/mm/rev), and torque penetration index (*TPI*, kN m/cutter/mm/rev) are selected as the TBM operation indicators:

(5) $$FPI=\frac{F_{n}}{num\times PR}$$

(6) $$TPI=\frac{T_{n}}{num\times PR}$$

where *F*_n_ is cutterhead load (kN), *P*_n_ is actual torque (kN), and *num* signifies the numbers of cutters on the cutterhead.

### 2.3. Stacking Ensemble-Learning Model and the Workflow

**Figure 3:**
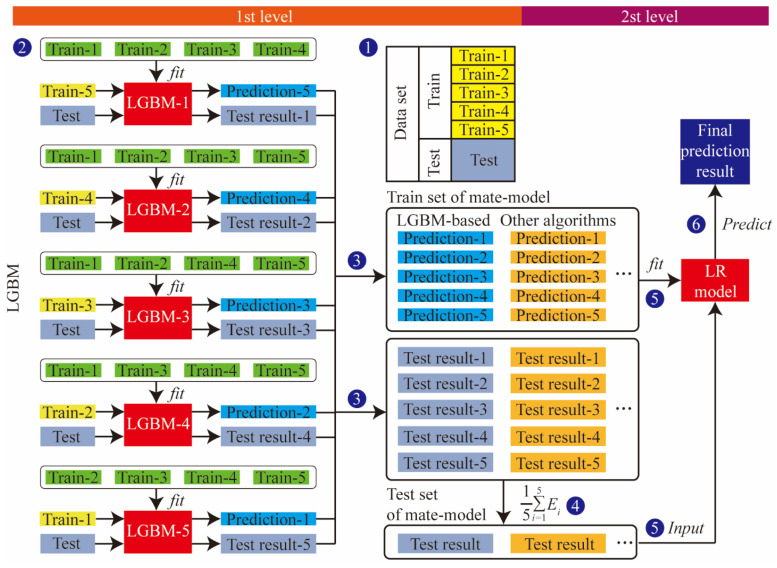
Stacking ensemble-learning model’s flowchart.

In order to improve the accuracy and stability, a two-layer stacking ensemble-learning model is developed. It can mine characteristic information more accurately than a single model. The workflow of the proposed method is shown in Figure 3.

The stacking ensemble learning first divides the original dataset *(a_n_*, *b_n_)* into a training set and a test set in a 4:1 ratio, where *a_n_* is the feature and *b_n_* is the label. In order to train and test the prediction ability, the training set is divided into K-parts through the K-Fold function. For example, when *K* = 5, the training set Train is divided into *Train-*1, *Train-*2, *Train-*3, *Train-*4, and *Train-*5. *Train_-k_* and *Train_k_* are defined as the K-th test set and training set in K-fold cross-validation, respectively. Five models were obtained after five cross-validations, as well as the prediction results, *Prediction-*1, *Prediction-*2, P*rediction-*3, *Prediction-*4, *Prediction-*5, for the test set after re-slicing on the five models. The length of the vertical stack of five predictions is identical to the Train length. The test set is brought into the five models to determine the prediction result (*test result)*, as shown in Figure 3.

(7) $$Train={Train}_{-K}+{Train}_{K},\;0<K\leq 5$$

The output of the first layer is used as the input data for the second layer of stacking, and the results are output by the model in the second layer. When multiple base models are used, the predictions are horizontally stitched as described above. In this way, the transformation of all data from input features to output labels is achieved.

### 2.4. Performance Metrics Introduction

The evaluation indicators in the classification model include *Accuracy*, *Precision*, *Recall*, *F1-score*, and confusion matrix. Taking the binary classification problem as an example, all events are divided into positive (*P*) and negative (*N*), and predicted events are classified as true (*T*) and false (*F*). In this way, four predictions of *TN*, *TP*, *FN,* and *FP* are generated.

*Accuracy* represents the proportion of the correct number of predictions in the total sample, but it is not sensitive to the problem of sample imbalance. Therefore, *Precision*, *Recall*, and *F1-score* are designed to evaluate the classification accuracy of positive and negative classes. *Precision* indicates the proportion of the number of the true-positive samples to all predicted positive samples. *Recall* is the probability of being predicted as a positive sample out of actual positive samples. *F*1*-score* denotes the composite metric of *Recall* and *Precision*, eliminating the one-sidedness of these two indices to a certain extent.

An indicator, *S_k_*, is also designed for evaluating the confidence of the prediction results. When the predicted probability of the classifier output class is much larger than that of other classes, the confidence of the classifier is high, and the *S_k_* is closer to 0. Moreover, it also reflects the stronger stability of the model. *Accuracy*, *Precision*, *Recall*, *F*1*-score*, and *S_k_* are defined as follows:

(8) $$Accuracy=\frac{TP+TN}{TP+FP+TN+FN}$$

(9) $$Precision=\frac{TP}{TP+FP}$$

(10) $$Recall=\frac{TP}{TP+FN}$$

(11) $$F1-score=2\times \frac{Precision\times Recall}{Precision+Recall}$$

(12) $$S_{k}=\sum_{i=1}^{n}\left|y_{i}-a_{i}\right|$$

## 3. Experiment and Datasets

**Figure 4:**
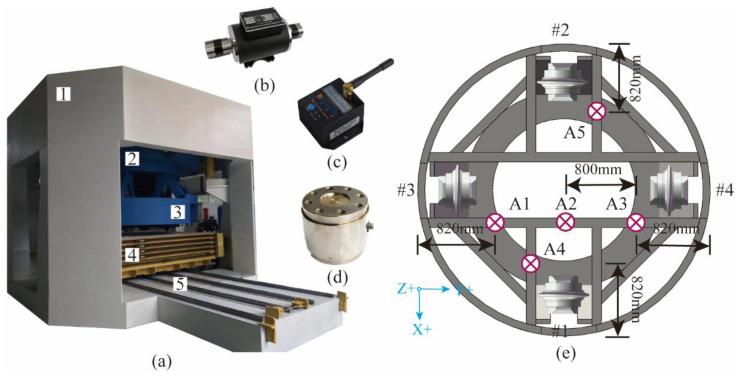
The full-scale rotary cutting machine (RCM): (**a**) 1—head frame; 2—movable frame; 3—cutterhead; 4—specimen box; and 5—guidance system. (**b**) Tension–compression force sensor. (**c**) Wireless vibration accelerometer. (**d**) Torque–speed sensor. (**e**) Cutterhead and A1–A5 monitoring positions of vibration accelerometer.

This experiment was designed to demonstrate and validate the developed Rock mass classification-forecasting method. The tests were conducted on a full-scale rotary cutting machine (RCM) in the State Key Laboratory of Shield Machin and Boring Technology, Zhengzhou, China, as shown in Figure 4. The deep neural networks evaluated in this research were applied using the open-source Pytorch. A computer with an Intel Core AMD Ryzen 7 4800 H 2.90 GHz 16-core CPU with 16 GB of memory running a 64-bit operation was used to train the model.

### 3.1. Experiment Setup

#### 3.1.1. Multi-Mode Boring Test System

The RCM is a comprehensive test facility for studying the rock-breaking mechanism of the TBM cutter [13]. RCM is mainly composed of the following parts: closed reaction frame, hydraulic propulsion and control system, spindle transmission system, spindle hydraulic and control system, main guide system, cutterhead and cutter box, rock box and guiding device, various types of sensor measurement devices, and computer measuring and data-processing systems. The disc cutter measured 18 inches, with a blade angle of 20 and a cutting-edge width of 0.9 inches. The main measurement methods include using a tension–compression force sensor to measure the thrust of the cutterhead; using a wireless vibration accelerometer to measure the vibration acceleration of the cutterhead in three directions (driving direction, cutterhead radial, and cutterhead tangential); measuring cutterhead advancing displacement, rotation speed, and torque by a wire-displacement sensor and torque–speed sensor. The detailed device profile is summarized in Table 2.

#### 3.1.2. Preparation of Rock Specimens

**Figure 5:**
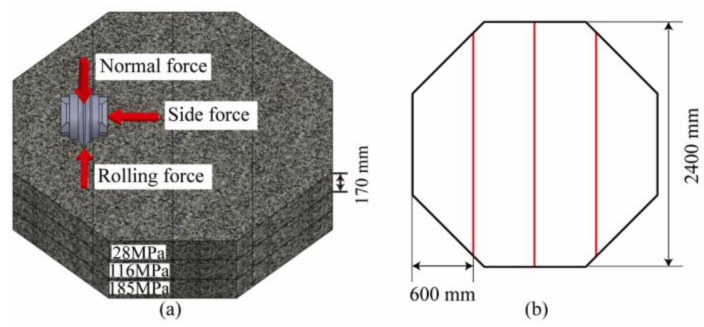
The rock samples: (**a**) layout and (**b**) size.

The shape of the rock specimen is a regular octagonal prism with a side length of 960 mm and a thickness of 550 mm. The thickness direction is composed of three layers of rocks with different strengths spliced, with uniaxial compressive strengths (UCS) of 28 Mpa (soft rock), 116 Mpa (medium–hard rock), and 185 Mpa (hard rock). In order to ensure that the integrity of the lower rock mass is not affected by the testing of the upper layer, the UCS value of the upper layer is always smaller than that of the lower layer. The size and layout of the rock samples are shown in Figure 5; the rocks were fixed in the specimen box using concrete grout.

#### 3.1.3. Testing Procedure

Since the surface of the rock sample is smooth and different from the tunnel surface, the sample must be pretreated before testing. The rock surface is slowly cut to form a series of equidistant grooves until the experimental monitoring data stabilize. Then, the operating parameters and cutting depth for each step are designed as listed in Table 3. The tests are represented as strings encoded in the following specific format. For example, in 1-4-4, the first number refers to the number of layers in the tunneling direction, which ranges from 1 to 18. The second number refers to the rotational speed (RS = 4 rev/min). The third number is the advance rate (AR= 4 mm/min). Each cut is made to a depth of 20 mm, and the slag is cleaned after each test. The rock is drilled in 6 passes of each strength in the direction of excavation. The radial distances from the installation position to the center of the cutterhead from cutter No. 1 to 4 are 726 mm, 826 mm, 926 mm, and 1026 mm. The inner rock is removed by a single cut after the No.1 cutter moves inward.

### 3.2. Statistics and Analysis of Datasets

#### 3.2.1. Datasets Division

**Figure 6:**
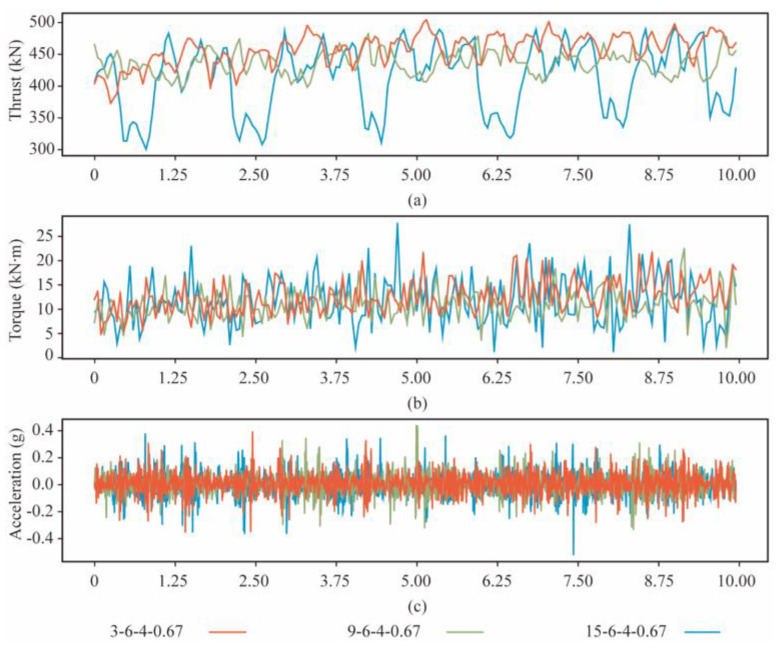
The response of the cutterhead at three strengths of rock mass. (**a**) Thrust. (**b**) Torque. (**c**) Acceleration.

A sliding sampling window is used to segment the table data to expand the dataset. The window and the overlap length are the time for 1 and 0.5 revolutions of the cutterhead, respectively. The dataset includes 273 samples of rocks in each strength. About 80% of the instances in each rock mass class are chosen as the training set, and the remaining instances are the test set. Figure 6 shows the time signals of thrust, torque, and vibration at different strengths of rock mass after data transformation (*R* = 6 rev/min, and *T* = 4 mm/min).

#### 3.2.2. Discussion on Distribution of the Filter Group

**Figure 7:**
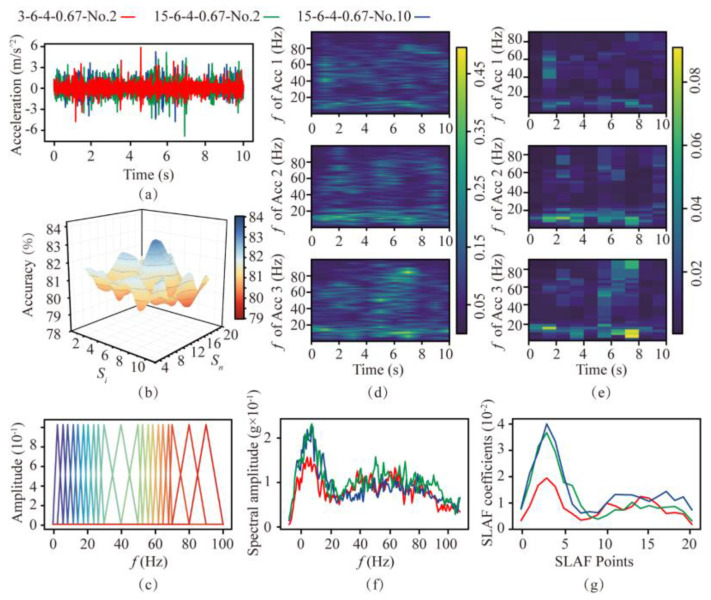
Comparison of spectrogram−based local amplification feature based on filters with different distributions. (**a**) Time-domain signals in soft and hard rock. (**b**) Accuracy under different filter banks. (**c**) Final filter bank used. (**d**) Spectrum of the three signals. (**e**) SLAF coefficients of the three signals. (**f**) Frequency−domain diagram. (**g**) SLAF coefficient diagram.

According to the SLAF the number and distribution of filter banks need to be determined, as they are important for the computational efficiency and accuracy of rock mass-classification prediction. In order to analyze the distinguishing effect of SLAF, the processing flow on soft and hard rocks is shown in Figure 7 (*R* = 6 rev/min, and *T* = 4 mm/min). Figure 7a shows the time-domain signals of the second rotation in soft rock (3-6-4-0.67-No.2, Acc 1) and the second and tenth rotation in hard rock (15−6-4-0.67-No.2, Acc 2, 15-6-4-0.67-No.10, Acc 3). The difference in amplitude of the cutterhead at each frequency is indicated by the coloring. It can be seen from Figure 7d,f that the spectrums of the rock at the same strength are similar, with the main frequencies concentrated in the ranges of 1–30 Hz and 50–70 Hz, suggesting that these two frequency ranges are important for rock mass-classification prediction.

Based on the above results, the filter bank in the main frequency range needs to be encrypted. The encryption interval is selected from 1, 2, 3, 4, and 5 Hz, and the remaining areas are selected from 5, 10, and 15 Hz. These intervals are tested against the RF algorithm via a grid search, and the test results are shown in Figure 7b. Finally, the interval is determined to be 3 Hz for the encrypted area and 5 Hz for the non-encrypted area. The distribution of the filter bank is shown in Figure 7c. The SLAF coefficient of the three signals obtained by the above method is shown in Figure 7e,g. It can be seen that the SLAF coefficient map has a contrasting relationship with the spectrogram, and its waveform is similar to the spectrum waveform. The main part is retained and highlighted, amplifying the gap in the main frequency range. The other parts are smoother in SLAF, which can be used to classify and identify rock mass strengths.

#### 3.2.3. Statistical Features of Data

**Figure 8:**
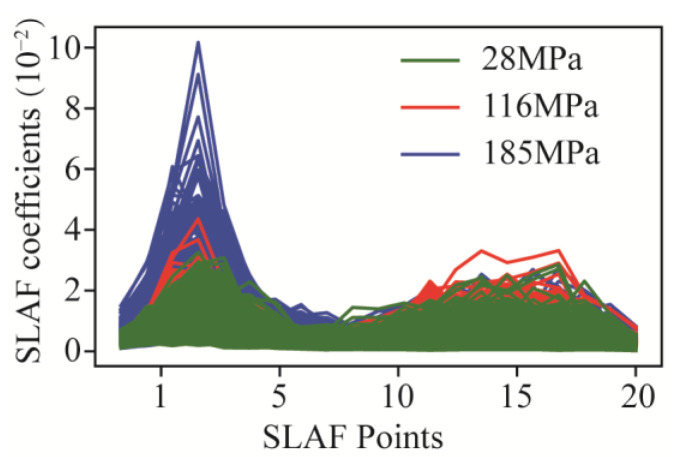
SLAF coefficients on three rock mass.

The results of experiment dataset are the primary analysis data for predicting. Table 4 offers a summary of the data statistics, including the mean, standard deviation, minimum, maximum, 25th percentile (Q1), 50th percentile (Q2), and 75th percentile (Q3). The naming rules of features are signal−type abbreviations (torque, T (kN m); thrust, F (kN); and vibration, A (m/s^2^)) and statistical method (subscript). Mean refers to the average of each feature that is used to observe the central tendency of the data. Standard deviation is a measure of the degree of variation in or dispersion of each part. And all the SLAF coefficients are shown in Figure 8.

**Figure 9:**
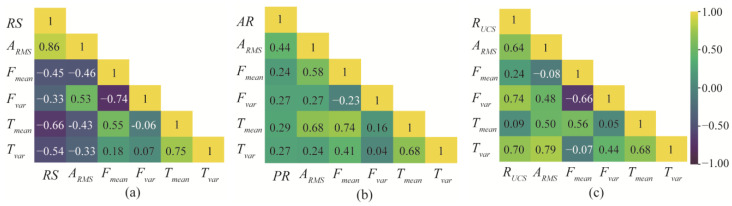
Pearson correlation coefficients: (**a**) rotating speed, (**b**) advance rate, and (**c**) rock strength. (Yellow indicates that the two variables are positively correlated, and purple indicates that the two variables are negatively correlated.)

The effect of rotational speed, advance rate, and rock mass strength changes on the thrust, torque, and vibration of the cutterhead are analyzed. The cutting tests are performed by varying only the rotational speed (*UCS* = 28 Mpa; *AR* = 6 mm/min; and *RS* = 4, 5, 6 rev/min), advance rate (*UCS* = 28 MPa; *RS* = 4 rev/min; and *AR* = 4, 5, 6 mm/min), and rock mass strength (*RS* = 4 rev/min; *AR* = 4 mm/min; and *UCS* = 28 MPa, 116 MPa, 185 MPa). Moreover, the size of thrust, torque, and vibration are synchronously monitored. Through these experiments, Pearson correlation coefficients between the five variables of vibration RMS, mean, and variance of thrust and torque are obtained, as shown in Figure 9.

#### 3.2.4. Pearson Correlation Coefficient

The covariance can explain the direction of correlation between two variables. However, the dimensions of each data feature are quite different. The Pearson correlation coefficient, γ, is obtained by dividing the covariance of the two variables by the standard deviation. According to the Cauchy–−Schwarz inequality, γ is between −1 and 1, with negative values representing a negative correlation and positive values representing a positive correlation. The Pearson correlation coefficient, γ, is defined as follows:

(13) $$\gamma =\frac{\sum_{i=1}^{n}\left(x_{i}-x)(y_{i}-y\right)}{\sqrt{\sum_{i=1}^{n}x_{i}-x}\sqrt{\sum_{i=1}^{n}y_{i}-y}},\;-1\leq \gamma \leq 1$$

It can be found that, as the rotational speed increases, the average amplitude and peak of thrust and torque gradually decrease, and the RMS value of vibration increases. With the increase in the advance rate, the mean and variance of the thrust and torque gradually increase, and the degree of vibration gradually increases. In addition, the mean value and variation range of thrust and torque, and the degree of vibration increase with the increasing rock strength.

## 4. Model

### 4.1. Model Selection and Combination

To build the stacking ensemble-learning model, three components must be defined: combinations of input base model features, combinations of base algorithms, and types of meta−model algorithms that combine them. Each signal type is weighted differently when predicting rock mass−strength classes, especially in the soft—hard stratum. All features are divided into four groups and input into different base models, including thrust feature, torque feature, vibration feature, and the combination of all signal features.

When selecting the base model, it is necessary to comprehensively consider the accuracy and the correlation. The model needs to reflect the advantages of the stacking ensemble-learning model, classifying the results from various spatial perspectives. Therefore, several models that have performed well in classification prediction are selected, including logistic regression (LR), SVM, k-nearest neighbor (KNN), RF, gradient-boosting decision tree (GBDT), extreme gradient boosting (XGBoost), light gradient-boosting machine (LGBM), categorical boosting (CatBoost), CNN, and LSTM. The correlation between the prediction results can be reflected by the Pearson correlation coefficient.

For the selection of the meta-model, simpler algorithms are often selected to prevent the overfitting of the stacking ensemble-learning models. Therefore, the meta-model used in this study is selected from LR, SVM, LGBM, and CatBoost.

### 4.2. Feature Importance Identification

XGBoost, GBDT, and RF can obtain feature contribution scores according to the gain of decision tree. In model training, the contribution scores are directly related to the usage efficiency of each feature. The importance of each feature is ranked using XGBoost, GBRT, and RF, and the top eight are shown in Table 5. Although the feature rankings of the three models are different, most features are of similar importance. The first two features are both *F_peak_* and *A_Q2_*, followed by the features that all appear in three models, which are *T_peak_*, *T_var_*, and *T_Q1_*. The variables that appear twice are *F_var_* and *A_Q3_*, and the variables that appear once are *A_65_* and *A_68_*.

### 4.3. Establishment of Stacking Ensemble-Learning Model

**Figure 10:**
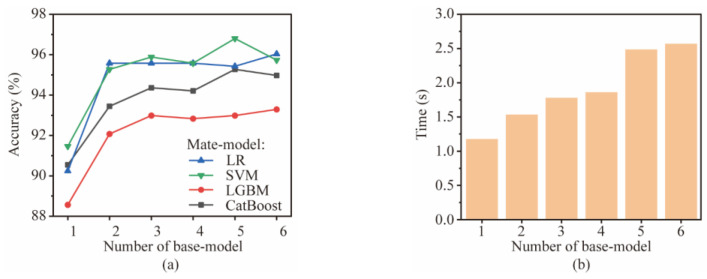
Performance comparison of different combinations: (**a**) accuracy and (**b**) time.

This section explores the optimal number of base models. The base model number affects the computational time and accuracy of the stacking ensemble-learning model. The number of base models trained on each signal type ranges from 0 to 6. The base models include LR, SVM, KNN, RF, GBDT, XGBoost, LGBM, CatBoost, CNN, and LSTM. The meta-model is selected from LR, SVM, LGBM, and CatBoost. The combination of optimal accuracy is selected via a grid search, and the base model is randomly selected. The final accuracy and computation time of the stacking ensemble-learning model are averaged over four runs, as shown in Figure 10.

It can be seen that, as the number of base models increases, the accuracy of the stacking ensemble-learning model shows a trend of first increasing sharply and then stabilizing. When the number of the base model is increased from 1 to 2, the accuracy increases by about 4%. When the number is 3 and above, the accuracy remains table. It also reflects that SVM has the best prediction effect as a meta-model. The computation time of the stacking ensemble-learning model increases with the number of the base model, with six base models being three times faster than one. Considering the computational time and accuracy, the base model number is finally determined to be 3.

In order to verify the influence of the base model combination on the prediction results, two random and diverse selection methods are used. The random selection only considers the accuracy of the algorithm used, while the diverse selection is conducted to ensure the differences between base models. Table 6 lists all combinations. For the selection of the meta-model, a simpler algorithm is selected to prevent the stacking ensemble-learning model from overfitting. The meta-model is selected from LR, SVM, LGBM, and CatBoost.

## 5. Results and Discussion

### 5.1. Accuracy

**Figure 11:**
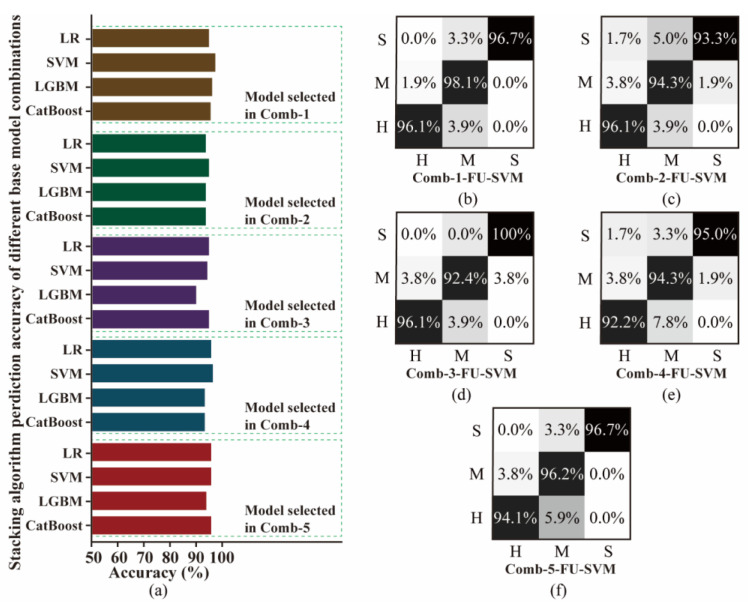
Comparison of the accuracy: (**a**) confusion matrix and (**b**–**f**) different combinations.

The accuracy of the stacking ensemble-learning model with the above combination is shown in Figure 11. The model from diverse selection (Comb-1) has higher accuracy than that from random selection. Base models with high accuracy can improve the predictive ability of the stacking ensemble-learning model. In addition, for different machine-learning algorithms, the essence is to observe the data differently and build corresponding models according to their respective logics. Therefore, the diversity and difference of base models allow for more accurate results.

**Figure 12:**
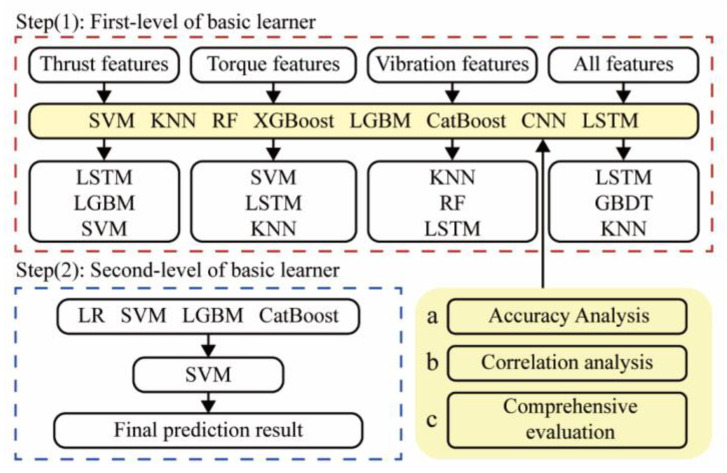
Rock mass-strength prediction based on diversified base models in the stacking ensemble-learning framework.

Based on the analysis results, the optimal combination is thrust features using LSTM, LGBM, and SVM algorithms; torque features using SVM, LSTM, and KNN algorithms; vibration features using KNN, RF, and CNN algorithms; and all signal features using LSTM, GBDT, and KNN algorithms. The accuracy of different meta-models is in the order of SVM, LR, GBDT, and LGBM, with the highest accuracy being 96.95%. The stacking ensemble-learning model combines the least-correlated base models, and the SVM meta-model has the highest accuracy. Therefore, the final base model in this study is Comb-1, and the meta-model is SVM. The combination method is shown in Figure 12 (Comb-1-FU-SVM).

Furthermore, the confusion matrix of five combinations of Comb-1-FU-SVM, Comb-2-FU-SVM, Comb-3-FU-SVM, Comb-4-FU-SVM, and Comb-5-FU-SVM are compared, as shown in Figure 11. It can be seen that Comb-1-FU-SVM has a relatively balanced accuracy of over 96% in classifying rock masses of S, M, and H strengths. Most of the prediction errors come from the misclassification of medium–hard rock as soft and hard, and there is no misprediction of soft rock into hard rock. This result indicates that the TBM signal features of medium–hard rock overlap with other rock mass strengths, making it difficult to optimally divide them. However, the constructed model has a strong predictive ability because it makes no inaccurate predictions for soft and hard rocks.

### 5.2. Stability

**Figure 13:**
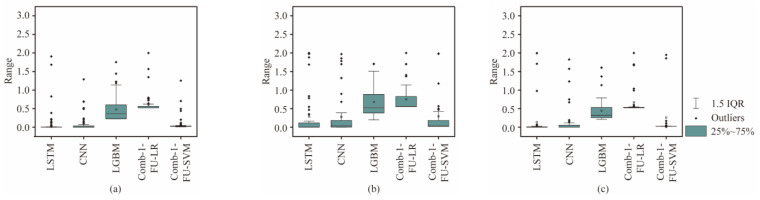
Comparison of the *S*_k_ range for predicting three rock masses with different strengths: (**a**) S, (**b**) M, and (**c**) H.

In order to verify the stability of the Comb-1-FU-SVM, four models were selected for comparison, namely LSTM, CNN, LGBM trained with all features, and the stacking ensemble-learning model Comb-1-LR. The quartiles of the stability, *S*_k_, are shown in Figure 13. As the mean of the interquartile range approaches 0, the stability of the model prediction is higher. In addition, the incorrectly predicted class is closer to the true class when the outlier is smaller.

Compared with other models, Comb-1-SVM has the smallest mean value and fluctuation range of *S*_k_. All indicators are concentrated in a small area, and the range of outliers is also small. It indicates that Comb-1-SVM has higher accuracy and confidence. Compared with soft and hard rocks, the predicted medium–hard rock has the largest interquartile range of *S*_k_, the highest average value, and a larger range of outliers. Therefore, when dealing with medium–hard rock, the Comb-1-SVM has low accuracy, a high error rate, and weaker stability in predicting rock mass strength.

## 6. Conclusions

In this paper, rock mass classification was studied based on full-scale rotary-cutting experiments. Thrust, torque, and vibration signals from TBM-equipped sensors were trained independently. A stacking ensemble-learning model was proposed using a novel spectrogram-based local amplification feature. The results indicate that the proposed model has high precision in mass classification prediction, which can be used to avoid disasters caused by mispredicting the strength of the rock mass. Some major conclusions can be derived:

(1) The peak value of thrust and the first dominant vibration frequency are the two most important features in model prediction.

(2) The mean and variance of thrust and torque and the root mean square of vibration positively correlate with rock strength.

(3) The number, type, and combination of base-models have a significant impact on the accuracy of the stacking ensemble-learning model.

(4) According to the traditional evaluation index and stability test index, the Comb-1-SVM has high accuracy and stability, which is suitable for rock mass classification prediction of the TBM tunnel face.

Due to the complex framework of the stacking ensemble-learning model, the basic model needs to be trained many times. It takes more time than a single model. Therefore, future studies will focus on building the appropriate distributed computing environment for different base models and reducing the algorithm’s complexity through multi-tasking. Our findings underscore the effectiveness of a stacking ensemble-learning model, featuring a novel spectrogram-based local amplification approach, in predicting rock mass properties with high precision. Nevertheless, the translation of this research into practical engineering applications necessitates extensive field-data collection and continuous model refinement. We are actively engaged in these endeavors and look forward to sharing our latest advancements and insights in the near future.

## Figures and Tables

**Table 1:** Types of features used by machine learning.

Feature	Formula	Feature	Formula
*Sign*	*x*_1_,*x*_2_,…,*x_n_*	*P_Q_*_1_	1 + (*n* − 1) × 0.25
*Max*	max(|*x*_1_|,|*x*_2_|, …,|*x_n_*|)	*P_Q_*_2_	1 + (*n* − 1) × 0.50
*Min*	min(|*x*_1_|,|*x*_2_|, …,|*x_n_*|)	*P_Q_*_3_	1 + (*n* − 1) × 0.75
*Peak*	max(|*x*_1_|,|*x*_2_|,…,|*x_n_*|) − min(|*x*_1_|,|*x*_2_|, …,|*x_n_*|)	*Mean*	$\frac{1}{n}\sum_{i=1}^{n}x^{i}$
*Var*	$\sqrt{\sum_{i=1}^{n}\frac{{\left(x_{i}-\mu \right)}^{2}}{N}}$	*RMS*	$\sqrt{\frac{1}{n}\sum_{i=1}^{n}x_{i}^{2}}$

**Table 2:** Basic specifications of the RCM.

Types	TBM General Properties	Size
Cutterhead operating parameter	Number of cutters	4 singles
	Rotational speed	0–8 rev/min
	Advance rate	0.05–50 mm/min
	Displacement	900 mm
Cutter sensor	Normal (thrust) force	0–500 kN
	Cutting forces	0–200 kN
	Side forces	0–200 kN
Vibration sensor	Range	0–10 g
	Frequency	0–2000 Hz

**Table 3:** Cutting parameters designed for each cutting step.

Test Coding	UCS/	VP_star_/	VP_end_/	AR/	RS/	PR/
	MPa	mm	mm	mm/min	rev/min	mm/rev
1-4-4	28	20	40	4	4	1.00
2-5-4		40	60	4	5	0.80
3-6-4		60	80	4	6	0.67
4-4-6		80	100	6	4	1.50
5-4-5		100	120	5	4	1.25
6-6-8		120	140	8	6	1.33
7-4-4	117	190	210	4	4	1.00
8-5-4		210	230	4	5	0.80
9-6-4		230	250	4	6	0.67
10-4-3		250	270	3	4	0.75
11-4-2		270	290	2	4	0.50
12-4-5		290	310	5	4	1.25
13-4-4	185	360	380	4	4	1.00
14-5-4		380	400	4	5	0.80
15-6-4		400	420	4	6	0.67
16-4-3		420	440	3	4	0.75
17-4-2		440	460	2	4	0.50
18-4-5		460	480	5	4	1.25

VP_star_: vertical position at the start of excavation. VP_end_: vertical position at the end of the excavation. AR: cutterhead advance rate. RS: rotational speed. PR: penetration rate.

**Table 4:** Statistical features of the dataset.

Features	Mean	Stdev	Min	Q1	Q2	Q3	Max
*FPI*	157.2	42.1	68.7	136.6	154.3	170.8	249.1
*TPI*	4.5	1.1	1.6	3.9	4.5	5.4	7.4
*A_peak_*	8.1	2.5	3.0	6.2	7.8	9.9	14.4
*A_mean_*	0.0	0.0	0.0	0.0	0.0	0.0	0.1
*A_var_*	0.5	0.3	0.1	0.2	0.4	0.7	1.1
*A_RMS_*	0.6	0.2	0.3	0.5	0.7	0.8	1.1
*A_Q1_*	0.3	0.1	0.6	0.4	0.3	0.2	0.1
*A_Q2_*	0.0	0.0	0.1	0.0	0.0	0.0	0.1
*A_Q3_*	0.3	0.1	0.1	0.2	0.3	0.5	0.6
*F_Peak_*	94.0	29.1	46.3	72.2	87.4	110.4	194.9
*F_mean_*	457.4	24.2	343.4	440.8	460.0	473.9	514.4
*F_var_*	721.5	529.3	151.7	359.6	554.0	901.4	3032.0
*F_RMS_*	458.2	24.0	345.0	441.3	460.6	474.6	515.1
*F_Q1_*	440.0	28.2	321.2	423.4	444.4	459.4	499.7
*F_Q2_*	459.3	24.1	338.3	442.0	462.5	477.0	517.5
*F_Q3_*	475.2	22.7	366.4	461.9	476.8	491.3	537.2
*T_Peak_*	25.5	11.6	9.3	17.7	22.7	32.2	62.6
*T_mean_*	13.3	2.3	7.8	11.5	12.9	14.8	19.6
*T_var_*	51.9	47.6	4.8	17.9	35.2	73.4	264.5
*T_RMS_*	14.9	3.1	8.6	12.4	14.4	17.1	23.2
*T_Q1_*	8.6	2.5	1.1	7.0	8.5	10.0	15.6
*T_Q2_*	12.8	2.4	7.2	11.1	12.3	14.4	22.2
*T_Q3_*	17.1	3.5	10.0	14.3	16.5	19.5	28.1

**Table 5:** Feature variable-importance analysis based on RF, GBDT, and XGBoost.

No.		1	2	3	4	5	6	7	8
RF	Features’	*F_peak_*	*A_Q2_*	*F_var_*	*A_Q3_*	*T_var_*	*T_peak_*	*A_65_*	*T_Q1_*
	Importance	0.15	0.12	0.12	0.06	0.06	0.05	0.05	0.05
GBDT	Features’	*F_peak_*	*A_Q2_*	*T_var_*	*T_Q1_*	*FPI*	*T_peak_*	*F_var_*	*A_Q3_*
	Importance	0.26	0.21	0.08	0.05	0.05	0.05	0.04	0.03
XGBoost	Features’	*F_peak_*	*A_Q2_*	*A_68_*	*T_peak_*	*TPI*	*T_Q1_*	*FPI*	*T_var_*
	Importance	0.29	0.28	0.16	0.07	0.07	0.05	0.05	0.05

**Table 6:** The combined form of the base model.

Name	Selection Type	Feature Type			
		Thrust	Torque	Vibration	All
Comb-1	Diverse	LSTM	SVM	KNN	LSTM
		LGBM	LSTM	RF	GBDT
		SVM	KNN	CNN	KNN
Comb-2	Random	LR	GBDT	KNN	KNN
		XGB	XGB	RF	LGBM
		LGBM	LGBM	GBDT	CatBoost
Comb-3	Random	LGBM	XGB	LR	LGBM
		GBDT	LR	SVM	SVM
		LSTM	CatBoost	LSTM	RF
Comb-4	Random	XGB	RF	CNN	LGBM
		CatBoost	CNN	GBDT	CNN
		GBDT	KNN	LGBM	CatBoost
Comb-5	Random	LGBM	LR	LSTM	KNN
		LSTM	RF	SVM	SVM
		CatBoost	XGB	CNN	GBDT

## Data Availability

Data will be made available on request.
